# Western Meteorology

**Published:** 1843-01

**Authors:** 


### THE WESTERN JOURNAL.

Vol. VII.—No. I.

LOUISVILLE, JANUARY 1, 1843.

### WESTERN METEOROLOGY.

While engaged, last summer, in a Medical survey of the valley of the Lakes, we made a visit to the *Western Reserve College* in the village of Hudson, Ohio, and became acquainted with Professor Loomis. This gentleman is already known to such of our readers as cultivate the science of Meteorology, one of the most charming and useful of the various handmaids of Medicine. But we fear, that the number who have enlisted her into their service, is not great. Would that we could augment it—could impress upon all, the manifold advantages they could derive, in their aetiological inquiries, from the theoretical and practical study of the atmosphere; in which are concealed so many floating *semina* of disease; and whose varying states of heat and moisture, exert on the human constitution such decided effects, both for good and evil. Professor Loomis has made an extensive collection of meteorological instruments, of the exactest kind; and is most punctual, accurate and persevering in his observations on the temperature and weight of the air, the course and force of the winds at the earth's surface; the direction, form and extent of the clouds, and quantity of rain; the state of the hygrometer, and the dew point. It is only by such observations, cotemporaneously made in various parts of our vast platform, from the Lakes to the Gulf, that the laws of our climate, and its influence in modifying our diseases, can be revealed. The professor showed us a respectable catalogue of collaborators, residing in various parts of the United States, with whom he exchanges printed transcripts of his observations, monthly; and assured us, that he would take great pleasure in augmenting the number of these exchanges. We hope this notice may be the means of adding many to his list. D.

